# Fatal Treatment of a Case of Tinea Capitis

**Published:** 1805-10-01

**Authors:** J. Weston

**Affiliations:** Shoreditch


### Fatal Treatment of a Case of Tinea Capitis; *Communicated by* Mr. J. Weston, *of Shoreditch.*

THOMAS MANN, aged eight years, who had long been afflicted with tinea capitis, which had obstinately resisted all the common remedies, had on Saturday, Aug. 3, the expressed juice of tobacco* applied over his head, by his father, who had been recommended to do so by a neighbour. The application was finished at five minutes before two o'clock, P. M. and he almost immediately afterwards complained of giddiness and loss of sight, so that his father smilingly said, "the boy is drunk." He soon after became sick, vomited frequently and in large quantity; he had also inclination to go to stool, but could not evacuate; his limbs tottered, his face was pale, and covered with a cold sweat; his mother assisted him up stairs to bed, into which he had no sooner entered than he had an involuntary dicharge of fæces. His countenance now appeared sunk; his limbs were motionless excepting that now and then his legs were drawn towards his belly convulsively. He spoke but seldom; but when he did utter any thing, it was to complain of an extreme degree of thirst and of violent pain in his bowels, which seemed distended with flatus. Still he vomited frequently, and the whole body was bedewed with a cold sweat; he gradually became weaker, and at half past five o'clock he expired, which was three hours and a half after the application of the poison.

### Dissection.

*Head.* The pericranium separated more easily than usual from the cranium, and some watery fluid was interposed between it and the skull. The membranes of the brain were healthy, except that there appeared to have been some slight effusion of lymph between the tunica arachnoides and pia mater; but this did not seem to have happened lately. The ventricles did not contain more than their proper quantity of water. The pineal gland had a hydatid in it.

The viscera of the abdomen were all in a natural state, excepting the small intestines, which appeared somewhat more loaded with blood than usual; all the viscera of the

* The dried tobacco leaf is wetted sufficiently to damp it, it is then put between iron plates, and pressed, by which means the juice is procured.

chest were perfectly sound; the blood in the heart was principally in a fluid state, but there was a coagulum in the right ventricle.

It appeared therefore by dissection that this boy's death was not attributable to any organic defect, but was to be ascribed entirely to the operation of an active vegetable poison upon the nervous system.

Several instances have been recorded of similar effects being produced by the unguarded use of this substance as an injection, and such cases cannot be too generally known, or too frequently related, as a knowledge of these facts will serve to put medical men upon their guard in using a remedy, which, without proper attention, is likely to destroy the life which it is intended it should preserve.

*August* 19, 1805.

